# Targeting prokineticin system counteracts hypersensitivity, neuroinflammation, and tissue damage in a mouse model of bortezomib-induced peripheral neuropathy

**DOI:** 10.1186/s12974-019-1461-0

**Published:** 2019-04-17

**Authors:** Giorgia Moschetti, Giada Amodeo, Daniela Maftei, Roberta Lattanzi, Patrizia Procacci, Patrizia Sartori, Gianfranco Balboni, Valentina Onnis, Vincenzo Conte, Alberto Panerai, Paola Sacerdote, Silvia Franchi

**Affiliations:** 1 Department of Pharmacological and Biomolecular Sciences, Università degli Studi di Milano, via Vanvitelli, 32, 20129 Milan, Italy; 2 Department of Biochemical Sciences “Alessandro Rossi Fanelli”, Sapienza University of Rome, Rome, Italy; 3 Department of Physiology and Pharmacology “Vittorio Erspamer”, Sapienza University of Rome, Rome, Italy; 4 Department of Biomedical Sciences for Health, Università degli Studi di Milano, Milan, Italy; 5 Department of Life and Environmental Sciences, Unit of Pharmaceutical, Pharmacological and Nutraceutical Sciences, University of Cagliari, Cagliari, Italy

**Keywords:** Prokineticins, Neuropathic pain, Bortezomib, Neuroinflammation, Macrophages

## Abstract

**Background:**

Neuropathy is a dose-limiting side effect of many chemotherapeutics, including bortezomib. The mechanisms underlying this condition are not fully elucidated even if a contribution of neuroinflammation was suggested. Here, we investigated the role of a chemokine family, the prokineticins (PKs), in the development of bortezomib-induced peripheral neuropathy (BIPN), and we used a PK receptor antagonist to counteract the development and progression of the pathology.

**Methods:**

Neuropathy was induced in male C57BL/6J mice by using a protocol capable to induce a detectable neuropathic phenotype limiting systemic side effects. The presence of allodynia (both mechanical and thermal) and thermal hyperalgesia was monitored over time. Mice were sacrificed at two different time points: 14 and 28 days after the first bortezomib (BTZ) injection. At these times, PK system activation (PK2 and PK-Rs), macrophage and glial activation markers, and cytokine production were evaluated in the main station involved in pain transmission (sciatic nerve, DRG, and spinal cord), and the effect of a PK receptors antagonist (PC1) on the same behavioral and biochemical parameters was assessed. Structural damage of DRG during BTZ treatment and an eventual protective effect of PC1 were also evaluated.

**Results:**

BTZ induces in mice a dose-related allodynia and hyperalgesia and a progressive structural damage to the DRG. We observed a precocious increase of macrophage activation markers and unbalance of pro- and anti-inflammatory cytokines in sciatic nerve and DRG together with an upregulation of GFAP in the spinal cord. At higher BTZ cumulative dose PK2 and PK receptors are upregulated in the PNS and in the spinal cord. The therapeutic treatment with the PK-R antagonist PC1 counteracts the development of allodynia and hyperalgesia, ameliorates the structural damage in the PNS, decreases the levels of activated macrophage markers, and prevents full neuroimmune activation in the spinal cord.

**Conclusions:**

PK system may be a strategical pharmacological target to counteract BTZ-induced peripheral neuropathy. Blocking PK2 activity reduces progressive BTZ toxicity in the DRG, reducing neuroinflammation and structural damage to DRG, and it may prevent spinal cord sensitization.

## Background

Chemotherapy-induced peripheral neuropathy (CIPN) represents a critical side effect of many chemotherapeutics, including bortezomib (BTZ), a first-generation proteasome inhibitor approved for the treatment of multiple myeloma [1, 2]. Neuropathy develops in about one third of patients undergoing BTZ, and it is characterized by the presence of spontaneous pain and mechanical hypersensitivity leading to dose reduction or treatment discontinuation. The molecular mechanisms underlying BTZ-induced peripheral neuropathy (BIPN) remain largely unclear even if the involvement of mitochondrial changes, oxidative stress, transient potential channels activation [3, 4], and in particular a role of neuroinflammation has been recently suggested for CIPN and specifically for BIPN development [5–8]. Penetration of chemotherapeutic agents into the central nervous system is relatively poor, whereas chemotherapeutics can cross the blood-nerve barrier, accumulating in the dorsal root ganglia (DRG) and peripheral nerve, exerting a toxic action and making these stations the main direct target of drug cytotoxicity. This condition leads to immune cell, in particular macrophages, infiltration and activation in these areas, promoting neuropathy progression [8–10]. We previously described a role of a recently discovered family of chemokines, the prokineticins (PKs), as modulators of immune function [11, 12] and key players in the development of experimental pathological pain [13, 14]. The prokineticin family includes two proteins: the mammalian prokineticin 1 (PK1 or endocrine gland derived vascular endothelial growth factor (EG-VEGF)) and PK2 (or mammalian-Bv8) and two G-protein receptors (PK-R1 and PK-R2), widely distributed in regions of the nervous system related to pain transmission and also expressed by immune cells [15]. We demonstrated that PKs are capable to induce a pro-inflammatory macrophage profile, stimulating chemotaxis and prompting the release of pro-inflammatory cytokines [16]. Moreover, PKs can directly induce hypersensitivity when injected in naïve mice [17–19]. Primary sensitive neurons co-express PKRs and the transient potential receptor vanilloid 1 (TRPV1) thus cooperating in nociceptor sensitization [15]. In addition, the activation of PK system in the peripheral nerves, DRG, and spinal cord correlates with the presence of neuroinflammation and the development of experimental neuropathic pain induced in mice by damage to the sciatic nerve [14, 20, 21] or by a metabolic disorder like diabetes [13]. In these experimental models, the treatment with antagonists of PK system was able to contrast or prevent the neuropathic symptomatology. On the basis of these considerations, our hypothesis is that PK system could represent a strategical target to counteract BIPN.

In this paper, we investigated the role of the PK system in the development of experimental BIPN and the therapeutic effect of PK-R antagonism. In order to do that, we evaluated in sciatic nerve, DRG, and spinal cord the activation of PK system and neuroinflammation during the pathology progression performing biochemical and ultrastructural analysis 14 days after the first BTZ treatment (half cumulative dose) and at the end of the chemotherapeutic schedule.

## Methods

### Ethic statements

All the procedures performed on animals were in compliance with the international policies (EEC council directive 86/609, OJ L 358, 1 Dec. 12, 1987; Guide for the Care and Use of Laboratory Animals, US National Research Council, 8th ed., 2011) and were approved by the Animal Care and Use Committee of the Italian Ministry of Health (permission number 709/ 2016 to SF). All efforts were made to reduce the number of animals used and to minimize animal suffering in accordance with the 3R principles.

### Animals

A total of 126 9-week-old C57BL/6J male mice (Charles River, Calco, Italy) were used in this study. Mice were housed with light/dark cycles of 12 h, temperature of 22 ± 2 °C, humidity of 55 ± 10%, and food and water ad libitum. Animals were assigned to cages (3 animals/cage) with the help of randomly generated numbers.

### BIPN induction and therapeutic treatment with the PK-Rs antagonist PC1

Bortezomib (BTZ) (LC Laboratories; Woburn, MA) was freshly prepared before each administration. BTZ was dissolved in dimethylsulfoxide (DMSO) with a concentration of 1 mg/ml and diluted in sterile 0.9% NaCl (saline) solution to a final concentration of 40 μg/ml [22], and it was intraperitoneally (i.p.) injected at the final dose of 0.4 mg/kg. For each cycle, BTZ (0.4 mg/kg) or vehicle were administered three times a week (every Monday, Wednesday, Friday) for a total of four consecutive weeks.

After verifying in mice the presence of mechanical and thermal hypersensitivity, the PKRs antagonist PC1 [23] was subcutaneously administered, in a therapeutic way, at the dose of 150 μg/kg [13], two times/day for 14 days, from day 14 until the end of the BTZ protocol (day 28). PC1 is a triazine-guanidine derivative that in vitro blocks PK2-induced intracellular Ca++ increase in PK-R1- and PK-R2-transfected CHO cells and in vivo antagonizes hyperalgesia induced by PK2. Affinity studies for PKRs receptors indicated a Ki of 22 nM and of 1610 nM for PK-R1 and PK-R2 respectively [23]. The chosen dose represents the most effective one contrasting pain as resulted from previously conducted dose-finding study performed in CCI neuropathic mice [14]. This dose was also effective in other neuropathic pain models like spared nerve injury model (SNI) [20] and in streptozotocin model of diabetic neuropathy [13].

### Experimental protocol: mechanical and heat thresholds determination

All the behavioral evaluations were performed by researchers who were blind to treatments. Behavioral evaluations were performed on both hind paws before starting BTZ protocol (0), 7, 14, 21 (corresponding to 7 days of chronic PC1 treatment), and 28 (corresponding to 14 days of chronic PC1 treatment) days after the first BTZ administration [13]. All measurements were done at least 14 h after the previous PC1 administration and before its first daily injection. The antiallodynic effect of a single PC1 injection was tested at the end of BTZ treatment (day 28), when hypersensitivity was maximal. Paw withdrawal thresholds were measured 30, 60, 120, 180, 210, and 240 min after PC1 injection.

### Double BTZ cycle

After a classical single BTZ cycle of 28 days, mice were monitored over time for the presence of allodynia, until they recovered to control values. At day 84, mice started a second BTZ cycle (using the before mentioned protocol) and their paw withdrawal thresholds were measured at the same time points as before, corresponding to 91, 98, 105, and 112 from the first BTZ administration.

### Mechanical allodynia

Mechanical allodynia was assessed using Dynamic Plantar Aesthesiometer [13] (Ugo Basile, Comerio, Italy). Animals were placed in a test cage with a wire mesh floor, and the rigid tip of a Von Frey filament (punctate stimulus) was applied to the skin of the mid-plantar surface of the hind paw with increasing force (ranging up to 10 g in 10 s), starting below the threshold of detection and increasing until the animal removed its paw. The withdrawal threshold was expressed in grams.

### Cold allodynia

Cold allodynia was evaluated as previously described [24]. Briefly, a drop (50 μl) of acetone was placed in the middle of the plantar surface of the hind paw. Mouse behavior was initially monitored for 20 s. If mice did not withdraw, flick, or stamp the hind paw within this time lapse, no other observations were made and the researcher assigned the score 0 to mice. However, if within this period the animal responded to the cooling effect of acetone, its behavior was assessed for an additional 20 s. Responses to acetone were graded, using a 4-point scale, as follows: 0, no response; 1, quick withdrawal, flick, or stamp of the paw; 2, prolonged withdrawal or repeated flicking (more than twice) of the paw; and 3, repeated flicking of the paw with licking directed at the plantar surface of the hind paw. Acetone was applied three times to each hind paw, and the responses were scored. Mean scores were then generated for each mouse.

### Thermal hyperalgesia

Thermal hyperalgesia was tested according to the Hargreaves procedure [25], slightly modified by us for mouse [14], using a Plantar test apparatus (Ugo Basile, Comerio, Italy). Briefly, mice were placed in small clear plexiglass cubicles and allowed to acclimatize. A constant intensity radiant heat source (beam diameter 0.5 cm and intensity 20 I.R.) was aimed at the mid-plantar area of the hind paw. Paw withdrawal latency (PWL), that is the time, in seconds (s), from initial heat source activation until paw withdrawal, was recorded.

### Biochemical, immunofluorescence, and electron microscopy evaluations

To evaluate the role of PK system and neuroinflammation in the development and progression of BTZ-induced peripheral neuropathy, mice were sacrificed at two different time points: 14 days after the first BTZ treatment (BTZ c.d. 2.4 mg/kg), before starting PC1 treatment; 28 days after the first BTZ treatment (BTZ c.d. 4.8 mg/kg), time point which corresponds to 14 days of PC1 chronic treatment. Mice were killed by CO_2_ inhalation for blood, lumbar spinal cord, DRG, and sciatic nerves collection. Nervous tissues were immediately frozen in liquid nitrogen and stored at − 80 °C until processing for mRNA extraction. Blood samples were centrifuged and serum was kept at − 20 °C until use.

All the evaluations and the subsequent statistical analysis were performed in a blind fashion.

### RNA extraction and real-time qPCR

Total RNA was isolated from sciatic nerves, DRG, and the lumbar spinal cords using TRIzol® Reagent (Invitrogen, ThermoFisher Scientific, Italy) according to the manufacturer’s instructions and re-suspended in 10–20 μl of RNase-free water. All procedures were performed as previously described in detail [13, 26]. Specific TaqMan probes/primers for mouse prokineticin receptors (Prokr1 Mm00517546_m1; Prokr2 Mm00769571_m1), cytokines (IL-1β Mm00434228_m1; IL-6 Mm00446190_m1; TNF-α Mm00443258_m1; IL-10 Mm00439616_m1), CD68 (Mm_03047343), TLR4 (Mm00445274_m1), and glyceraldehydes-3-phosphate dehydrogenase (GAPDH Mm99999915_g1) were purchased from Applied Biosystems. Threshold cycle numbers (Ct) of the specific gene of interest and the endogenous control gene GAPDH were determined by ABI PRISM 7000 Sequence Detection System.

The Ct value of the specific gene of interest was normalized to the Ct value of the endogenous control, GAPDH, and the comparative Ct method (2^−ΔΔCt^) was then applied using the control group (vehicle treated mice) as calibrator.

### Immunofluorescence

L4-L5 spinal cord, DRG, and sciatic nerve were dissected from transcardially perfused mice (PBS followed by 4% paraformaldehyde (PFA)), post-fixed in 4% PFA for 24 h, cryoprotected in 30% sucrose solution, embedded in cryostat medium, and frozen and cut using a cryostat. Prior to immunofluorescence staining, all sections were blocked with 3% normal donkey serum, containing 0.1% Triton X-100 for 30 min at room temperature. Spinal cord transverse sections (40 μm, free-floating) were incubated at 4 °C for 48 h, whereas DRG and sciatic nerve sections (20 μm, mounted on slides) were incubated at 4 °C overnight with the following primary antibodies diluted in PBS-0.3% Triton X-100: anti-PK2 (rabbit, 1:200, AbCam, Cambridge, UK), anti-PK-R1 and anti-PK-R2 (rabbit, 1:200, Alomone labs, Jerusalem, Israel), anti-GFAP (mouse, 1:400, Immunological Sciences, Italy), and anti-CD68 (mouse, 1:400, AbCam, Cambridge, UK) [14]. After washing, sections were incubated for 2 h at room temperature with anti-species IgG secondary antibodies coupled to Alexa Fluor®-488 or 555 (1:200, Immunological Sciences). Nuclei were stained with DAPI (1:500, Sigma Aldrich). Possible non-specific labeling of secondary antibodies was detected using secondary antibody alone. Images of stained sections were acquired using a laser-scanning confocal microscope (Leica SP5, Leica Microsystems, Wetzlar, Germany) connected to a digital camera diagnostic instrument operated by I.A.S. software of Delta Systems Italia (Milan, Italy) [14].

### Quantitative image analysis

To quantify the immunofluorescence positive area of CD68, PK2, and GFAP in the sciatic nerve and DRG, high-magnification images were captured with a × 40 objective at zoom factor 1 using a constant set of acquisition parameters. Six sections were captured from each of five animals per group. The analysis was performed using ImageJ software (version 1.47, http://imagej.nih.gov/ij/index.html, free software) within three boxes of 10^4^ μm^2^ per section, and a mean value was obtained by combining values from all three boxes.

To quantify the immunofluorescence positive area of CD68, PK2, and GFAP in the spinal cord, six L4-L6 immunofluorescence high-magnification images of the dorsal horns were captured as described above, from each of five animals per group. Quantification was performed within three boxes of 10^4^ μm^2^ per section that were placed in the lateral, central, and medial areas of the dorsal horns, and a mean value was obtained by combining values from the three boxes.

### Ultrastructural evaluations of DRG (electron microscope)

Mice were anesthetized (ketamine/xylazine i.p.) and transcardially perfused with a fixative solution (2% paraformaldehyde, 2% glutaraldehyde in cacodylate buffer, pH 7.3). From each animal, DRG (L4 and L5) were removed and immersed in the same fixative overnight at 4 °C. Subsequently, samples were washed in 0.2 M cacodylate buffer, postfixed in 2% OsO_4_ (Sigma-Aldrich) in the same buffer, washed in distilled water, and stained with 2% aqueous uranyl acetate. Then, it was carried out the dehydration in ethyl alcohol and embedded in Epon-Araldite resin.

Semithin sections (0.5 μm thick) of each DRG were stained with 0.5% toluidine blue in 1% sodium borate and examined with a light microscope (Zeiss Axiophot) for preliminary observations. Ultrathin sections (50 to 70 nm thick), cut on a Leica Supernova ultramicrotome, were stained with lead citrate and examined under a Zeiss EM10 electron microscope (Gottingen, Germany).

### ELISA

Serum was obtained by centrifuging blood samples 14 and 28 days after the first BTZ treatment. Prokineticin 2 levels were measured in mice serum by means of CSB-EL018747MO ELISA kit (Cusabio). Each sample was tested in double. Kit sensitivity: minimum detectable dose of mouse prokineticin 2 is 3.12 pg/ml.

### Statistical analysis

Experiments were designed to minimize the number of animals based on the results obtained in our previous studies [13, 21] and on pre-study power analysis considering the antiallodynic response as primary endpoint. Data are expressed as mean ± SD (six animals/group for behavioral and biochemical evaluations)

Statistical analysis was performed as described below:

- Data from behavioral analysis were analyzed by mean of two-way ANOVA analysis of variance followed by Bonferroni’s test for between-group comparisons in the post hoc analysis.
- For biochemical evaluations, statistical analysis was performed at day 14 by using *t* test and at day 28 by mean of one-way ANOVA followed by Bonferroni’s test for multiple comparisons. Differences were considered significant at *p* < 0.05. All the statistical analyses were performed using GraphPad 6 software (San Diego, CA).

## Results

The dose of BTZ was chosen from the literature [22] in order to minimize unspecific systemic toxicity. BTZ treatment was well tolerated by animals; no mice died or were in sufferance, maintaining explorative, grooming, and feeding activities comparable to those of control mice. BTZ did not induce a significant loss of weight in the animals (data not shown).

### BIPN development and effect of the PK antagonism

**Fig. 1:**
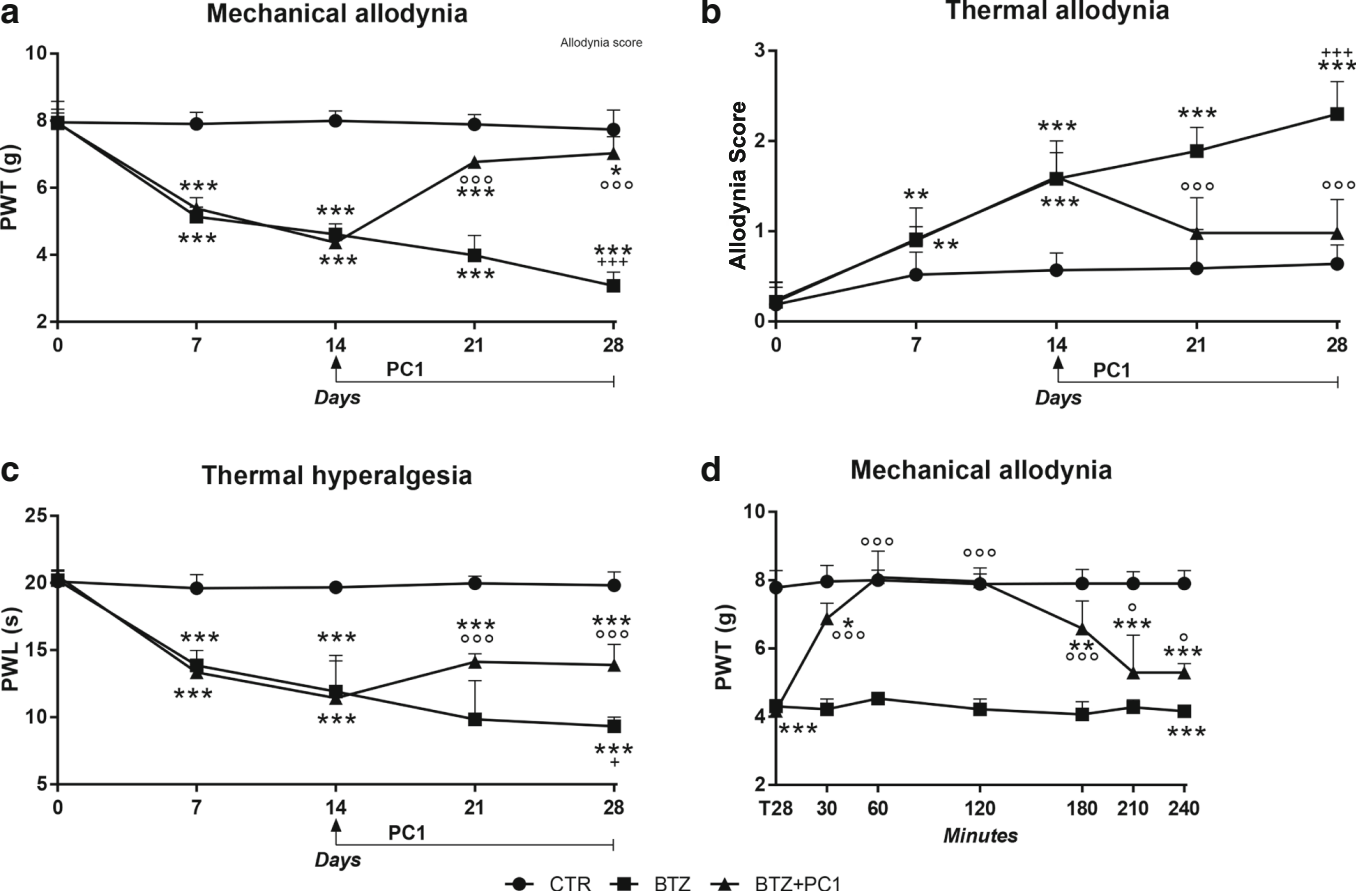
Anti-allodynic and anti-hyperalgesic effect of the PK-R antagonist PC1. **a**–**c** The effect of chronic PC1 on mechanical (**a**) and thermal (**b**) allodynia and on thermal hyperalgesia (**c**) which develop in mice after bortezomib (BTZ) treatment (0.4 mg/kg 3 times week/4 weeks). PC1 was administered (s.c. 150 μg/kg twice daily) for 14 days starting from day 14 (established hypersensitivity) until day 28. **d** The effect of a single PC1 injection (s.c. 150 μg/kg) performed at the end of BTZ protocol (day 28), when hypersensitivity was maximal. Paw withdrawal thresholds were measured 30, 60, 120, 180, 210, and 240 min after PC1 injection. Data represent mean ± SD of 6 mice/group. Statistical analysis was performed by mean of two-way ANOVA followed by Bonferroni’s post-test. *p < 0.05, **p < 0.01, ***p < 0.001 vs vehicle/CTR; °*p* < 0.05, °°°*p* < 0.001 vs BTZ; +*p* < 0.05, +++*p* < 0.001 vs BTZ mice at day 14 (before starting PC1 treatment)

As shown in Fig. 1, BTZ induces in mice a dose-related mechanical and thermal hypersensitivity characterized by the presence of allodynia (panels a and b) and hyperalgesia (panel c). Fourteen days after the first BTZ administration (BTZ cumulative dose, c.d., 2.4 mg/kg), mechanical and thermal thresholds of BTZ mice were already lowered if compared to those of the control mice (vehicle treated; *** *p* < 0.001 vs CTR), and a further decrease was evident at the end of the BTZ protocol at day 28 (BTZ c.d. 4.8 mg/kg; +++ *p* < 0.001 vs BTZ day 14). Treatment with the PK-Rs antagonist PC1 was started at day 14, in the presence of a well-established allodynia and hyperalgesia. PC1 was injected two times/day from day 14 until day 28, simultaneously to the BTZ treatment. After 7 days of chronic treatment (day 21), the antagonist can efficiently counteract both mechanical (panel a) and thermal allodynia (panel b) as well as thermal hyperalgesia (panel c), and its effect is maintained for the entire duration of treatment (°°°*p* < 0.001 vs BTZ).

We also evaluated the acute effect of a single PC1 injection on mechanical allodynia at the end of the BTZ protocol, when hypersensitivity was maximal (panel d). PC1 was able to rapidly counteract mechanical allodynia, and its effect was maximal between 60 and 120 min and then progressively diminished, although a significant difference from BTZ mice was still present 240 min after its administration.

#### PK and neuroimmune activation in peripheral and central nervous system

The activation of PK system and the presence of neuroinflammation were evaluated in the main stations of pain transmission (sciatic nerve, DRG, and spinal cord) 14 days after the first BTZ injection (corresponding to BTZ cumulative dose of 2.4 mg/kg) and the end of BTZ protocol (corresponding to BTZ cumulative dose of 4.8 mg/kg).

#### Peripheral nervous system

##### PK system (PK2 and its receptors) in the sciatic nerve

**Fig. 2:**
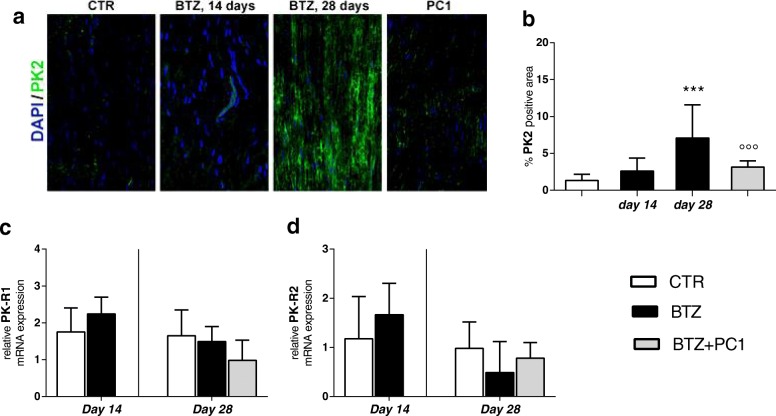
PK system activation in the sciatic nerve. **a** Representative images of PK2 immunofluorescence signal in the sciatic nerve sections of CTR, BTZ 14 days (corresponding to c.d. 2.4 mg/kg), BTZ 28 days (corresponding to c.d. 4.8 mg/kg), and PC1 (BTZ + PC1) mice. Cell nuclei were counterstained with DAPI (blue fluorescence). Quantitative analysis of PK2 signal (**b**) was computed as integrated optical density for arbitrary areas (6 sections per animal, 5 animals per group). One-way ANOVA was used for statistical evaluation, followed by Bonferroni’s test for multiple comparisons. ***p < 0.001 vs CTR; °°°*p* < 0.001 vs BTZ day 28. **c**, **d** mRNA levels of PK-R1 and PK-R2 respectively, measured by real-time qPCR, 14 days after the first BTZ administration (c.d. 2,4 mg/kg) and at the end of BTZ protocol (c.d. 4.8 mg/kg, day 28). Data represent mean ± SD of 6 mice/group. Statistical analysis was performed by mean of one-way ANOVA

Levels of PK2 were assessed by immunofluorescence staining. As reported in Fig. 2a, after 14 days of BTZ treatment, we did not observe any change of PK2, as demonstrated by the quantification of the percentage of PK2 positive area (panel b). At the end of the chemotherapeutic schedule (day 28), PK2 levels were increased, as shown by the representative immunofluorescence images (panel a) and by the quantification of PK2 positive area (panel b; ***p < 0.001 vs CTR). The treatment with PC1 can significantly reduce PK2 signal (panels a and b; °°°*p* < 0.001 vs BTZ day 28). PK-R1 and PK-R2 expression levels were never affected by BTZ treatment (panels c and d).

##### Effect of the PK-R antagonism on sciatic nerve neuroinflammation

**Fig. 3:**
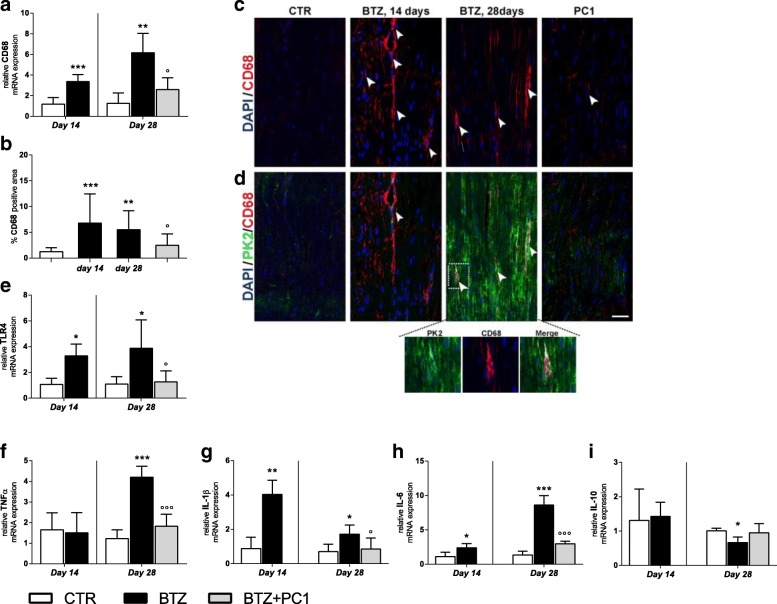
Effect of PC1 treatment on macrophage activation and cytokine levels in the sciatic nerve. **a**, **b** mRNA levels of CD68 and the percentage of CD68 positive area in the sciatic nerve of CTR, BTZ 14 days (day 14 corresponding to c.d. 2,4 mg/kg), BTZ 28 days (day 28, corresponding to c.d. 4,8 mg/kg), and PC1 (BTZ + PC1) mice. Quantitative analysis of CD68 signal was computed as integrated optical density for arbitrary areas (6 sections per animal, 5 animals per group). **c** Representative immunofluorescence images of CD68 in the sciatic nerve sections of the same experimental groups. One-way ANOVA was used for statistical evaluation, followed by Bonferroni’s test for multiple comparisons. **p < 0.01, ***p < 0.001 vs CTR; °*p* < 0.05 vs BTZ day 28. **d** Immunofluorescence double-staining shows the colocalization (yellow, arrowhead) of PK2 (green) with CD68 (activated macrophages, red) in the sciatic nerve of CTR, BTZ day 14, BTZ day 28, and BTZ + PC1 mice (PC1). Cell nuclei were counterstained with DAPI (blue fluorescence). **e**–**h** The mRNA levels of TLR4 and of the pro-inflammatory cytokines TNF-α, IL-1β, and IL-6 respectively. **i** The mRNA of the anti-inflammatory cytokine IL-10. All the measurements were performed 14 days after the first BTZ administration (c.d. 2.4 mg/kg), before starting PC1 treatment (CTR and BTZ groups), and at the end of the BTZ protocol (c.d. 4.8 mg/kg) (CTR, BTZ, BTZ + PC1 groups). mRNA levels, determined by real-time qPCR, were expressed in relation to GAPDH and presented as fold-increases over the levels of CTR animals (at the same time point). (**a**, **e**–**i**) Data represent mean ± SD of 6 mice/group. At day 14, statistical analysis was performed by means of *t* test while at day 28 by using one-way ANOVA followed by Bonferroni’s post-test. *p < 0.05, **p < 0.01, ***p < 0.001 vs vehicle/CTR (at the same time point); °*p* < 0.05, °°°*p* < 0.001 vs BTZ day 28

As illustrated in Fig. 3, at day 14, before starting PC1, we measured an increase of CD68 both as mRNA (panel a; ***p < 0.001 vs CTR) and as protein as reported in the immunofluorescence images of panel c and its quantification as percentage of CD68 positive area (panel b; ***p < 0.001 vs CTR). Simultaneously (day 14), we observed increased mRNA levels of TLR4 (panel e; *p < 0.05 vs CTR) and of pro-inflammatory cytokines IL-1β (panel g; **p < 0.01 vs CTR) and IL-6 (panel h;*p < 0.05 vs CTR) without changes in TNF-α (panel f) and in the anti-inflammatory cytokine IL-10 (panel i). By increasing the cumulative dose of BTZ at day 28, levels of CD68 (panels a, b, and c; **p < 0.01 vs CTR) and of TLR4 (panel e; *p < 0.05 vs CTR) were still upregulated. Moreover, at this time, we observed an increase in the levels of all the evaluated pro-inflammatory cytokines: TNF-α, IL-1β, IL-6 (panels f, g, and h respectively), and a decrease of IL-10 expression (panel i;*p < 0.05 vs CTR). Immunofluorescence double-staining images (panel d) illustrate that PK2 immunoreactivity partially colocalizes with the CD68 signal (panel d enlargement). PC1 treatment was able to contrast macrophage recruitment and activation, as demonstrated by the reduced levels of CD68 (panels a, b, and c; °*p* < 0.05 vs BTZ day 28) and TLR4 levels (panel e; °*p* < 0.05 vs BTZ day 28), to prevent the increase of the TNF-α (panel f) and the decrease of IL-10 (panel i) and to reduce the increased levels of IL-1β and IL-6 (panel g; °*p* < 0.05 vs BTZ day 28 and panel h; °°°*p* < 0.001 vs BTZ day 28). Consistent with what was already reported and quantified in Fig. 2a and b, PK2-positive immunoreactivity is lost in PC1-treated animals (panel d).

##### PK system in DRG

**Fig. 4:**
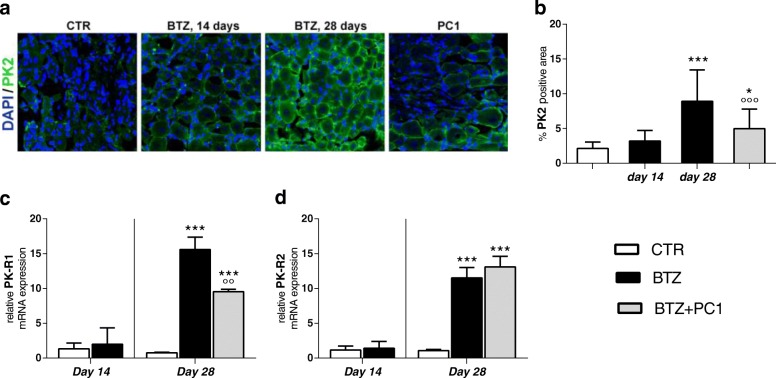
PK system activation in DRG. **a** Representative images of PK2 immunofluorescence signal in DRG sections of CTR, BTZ 14 days, BTZ 28 days, and PC1 (BTZ + PC1) mice. Quantitative analysis of PK2 signal (**b**) was computed as integrated optical density for arbitrary areas (6 sections per animal, 5 animals per group). One-way ANOVA was used for statistical evaluation, followed by Bonferroni’s test for multiple comparisons. *p < 0.05, ***p < 0.001 vs CTR; °°°*p* < 0.001 vs BTZ day 28. **c**, **d** mRNA levels of PK-R1 and PK-R2 respectively, measured by real-time qPCR, 14 days after the first BTZ administration (c.d. 2.4 mg/kg) in CTR and BTZ mice and at the end of the BTZ protocol (c.d. 4,8 mg/kg) in CTR, BTZ, and BTZ + PC1. mRNA levels, determined by real-time qPCR, were expressed in relation to GAPDH and presented as fold increases over the levels of CTR animals (at the same time point). Data represent mean ± SD of 6 mice/group. At day 14, statistical analysis was performed by means of *t* test while at day 28 by one-way ANOVA followed by Bonferroni’s post-test. ***p < 0.001 vs vehicle/CTR (at the same time point); °°*p* < 0.01 vs BTZ day 28

As illustrated in Fig. 4, at day 14, we did not observe any significant change in PK system in DRG. PK2 immunoreactivity is reported in panel a and quantified in panel b, while PK-R1 and PK-R2 are reported as mRNA in panels c and d respectively. However, at the higher BTZ cumulative dose, we observed a consistent increase of PK2 signal as shown by immunofluorescence representative images (panel a) and by the increase of percentage of PK2 positive area (panel b;*** *p* < 0.001 vs CTR), as well as an upregulation of both PK-R1 and PK-R2 mRNA levels (panel c and d; *** *p* < 0.001 vs CTR). PC1 treatment was able to contrast the increase of both PK2 (panels a and b; °°°*p* < 0.001 vs BTZ day 28) and PK-R1 (panel c; °°*p* < 0.01 vs BTZ day 28).

##### Effect of the PK-Rs antagonism on DRG neuroinflammation

**Fig. 5:**
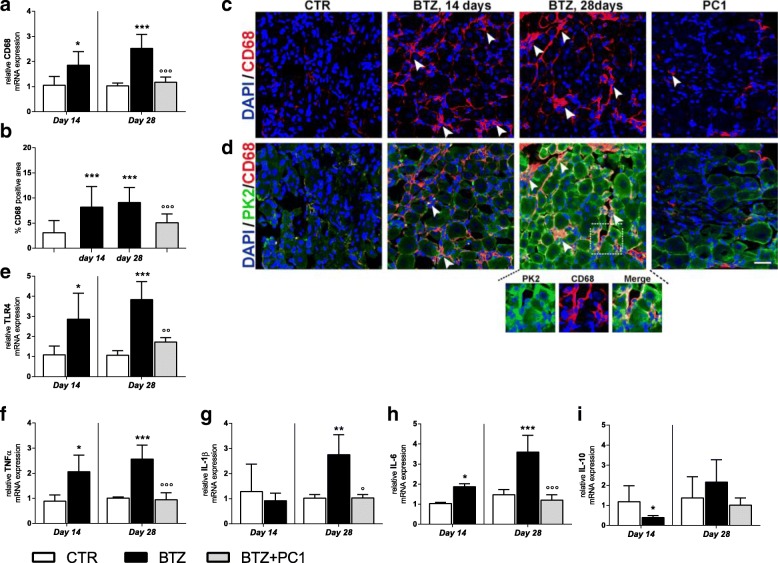
Effect of PC1 chronic treatment on macrophage activation and cytokine levels in DRG. **a**, **b** mRNA levels of CD68 and the percentage of CD68 positive area in DRG of CTR, BTZ 14 days (corresponding to c.d. 2.4 mg/kg), BTZ 28 days (corresponding to c.d. 4.8 mg/kg), and PC1 (BTZ + PC1) mice. Quantitative analysis of CD68 signal was computed as integrated optical density for arbitrary areas (6 sections per animal, 5 animals per group). One-way ANOVA was used for statistical evaluation, followed by Bonferroni’s test for multiple comparisons. ***p < 0.001 vs CTR; °°°*p* < 0.001 vs BTZ day 28. **c** Representative images of CD68 immunofluorescence signal in DRG sections while **d** immunofluorescence double staining images shows the colocalization (white arrowhead) of PK2 (green) with CD68 (activated macrophages, red) in CTR, BTZ 14 days, BTZ 28 days, and PC1 (BTZ + PC1) mice. **e**–**h** the mRNA levels of TLR4 and of the pro-inflammatory cytokines TNF-α, IL-1β, and IL-6. **i** The anti-inflammatory cytokine IL-10. All the measurements were performed 14 days after the first BTZ administration, before starting PC1 treatment (CTR and BTZ groups), and at the end BTZ/BTZ + PC1 protocol (CTR, BTZ, BTZ + PC1 groups). (**a**, **e**–**i**) Data represent mean ± SD of 6 mice/group. At day 14, statistical analysis was performed by means of *t* test while at day 28 by means of one-way ANOVA analysis of variance followed by Bonferroni’s post-test. *p < 0.05, **p < 0.01, ***p < 0.001 vs vehicle/CTR; °*p* < 0.05,°°*p* < 0.01, °°°*p* < 0.001 vs BTZ day 28

BTZ promotes also in DRG, a rapid macrophage recruitment and activation already evident at day 14. At this time point, as shown in Fig. 5, we observed in DRG sections increased levels of CD68 mRNA (panel a; *p < 0.05 vs CTR) and CD68 protein as reported by immunofluorescence pictures (panel c) and the quantification of percentage of CD68 positive area (panel b; ***p < 0.001 vs CTR). Simultaneously, we observed an increase of TLR4 mRNA (panel e; *p < 0.05 vs CTR) and a pro-inflammatory cytokine profile due to increased mRNA levels of the pro-inflammatory cytokines TNF-α (panel f; *p < 0.05 vs CTR) and IL-6 (panel h; *p < 0.05 vs CTR) and a decrease of the anti-inflammatory cytokine IL-10 (panel i; *p < 0.05 vs CTR). This pattern is also evident at day 28 when also IL-1β (panel g; **p < 0.01 vs CTR) is upregulated while no significant changes were observed for IL-10 (panel i) at this time. Immunofluorescence double-staining images illustrate that CD68 signal colocalizes in part with PK2 signal (panel d enlargement). PC1 treatment was able to normalize the levels of CD68 (panels a, b, and c; °°° *p* < 0.001 vs BTZ day 28), of TLR4 (panel e; °°*p* < 0.001 vs BTZ day 28), and of all pro-inflammatory cytokines investigated. As expected (see also Fig. 4), PK2 activation was switched off in PC1-treated mice and the association with CD68 was not present anymore.

#### Central nervous system

##### PK system in the spinal cord

**Fig. 6:**
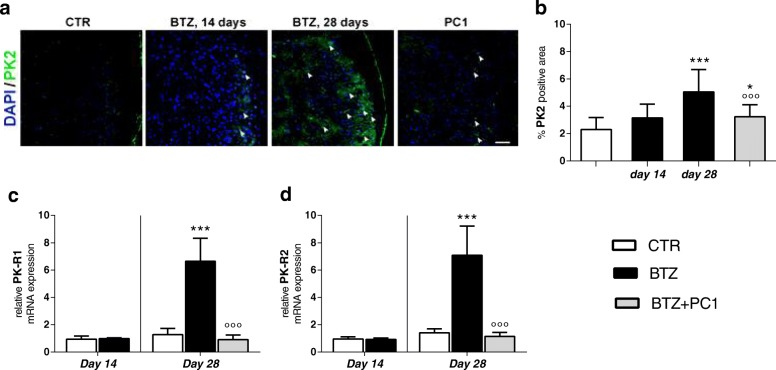
PK system activation in the spinal cord. **a** Representative images of PK2 immunofluorescence signal in spinal cord sections of CTR, BTZ 14 days (corresponding to c.d. 2,4 mg/kg), BTZ 28 days (corresponding to c.d. 4.8 mg/kg), and PC1 (BTZ + PC1) mice. Quantitative analysis of PK2 signal (**b**) was computed as integrated optical density for arbitrary areas (6 sections per animal, 5 animals per group). One-way ANOVA was used for statistical evaluation, followed by Bonferroni’s test for multiple comparisons. *p < 0.05, ***p < 0.001 vs CTR; °°°*p* < 0.001 BTZ day 28. **c**, **d** mRNA levels of PK-R1 and PK-R2 respectively, measured by real-time qPCR, 14 days after the first BTZ administration (CTR and BTZ) mice and at the end of the BTZ protocol (c.d. 4,8 mg/kg) in CTR, BTZ, and BTZ + PC1. mRNA levels, determined by real-time qPCR, were expressed in relation to GAPDH and presented as fold increases over the levels of CTR animals (at the same time point). Data represent mean ± SD of 6 mice/group. At day 14, statistical analysis was performed by means of *t* test while at day 28 by means of one-way ANOVA followed by Bonferroni’s post-test. ***p < 0.001 vs CTR; °°°*p* < 0.001 vs BTZ day 28

As described above for the PNS, also in the spinal cord 14 days after the first BTZ treatment, both PK2 (Fig. 6a, b) and PK-Rs receptor (Fig. 6c, d) levels appear comparable to those of the control mice. At the end of the chemotherapeutic schedule (day 28), BTZ increases PK2 signal in the dorsal horns of the spinal cord, as shown in the immunofluorescence pictures (panel a) and quantification of percentage of PK2 positive area (panel b; ***p < 0.001 vs CTR). An increase of PK-R1 and PK-R2 receptors (panels c and d; ***p < 0.001 vs CTR) was also present at this time point. Therapeutic treatment with PC1 was able to contrast PK system (PK2 and PK-R receptors) increase in the spinal cord (panels a, b, c, and d; °°°*p* < 0.001 vs BTZ day 28).

##### Effect of PK antagonism on spinal cord neuroinflammation

**Fig. 7:**
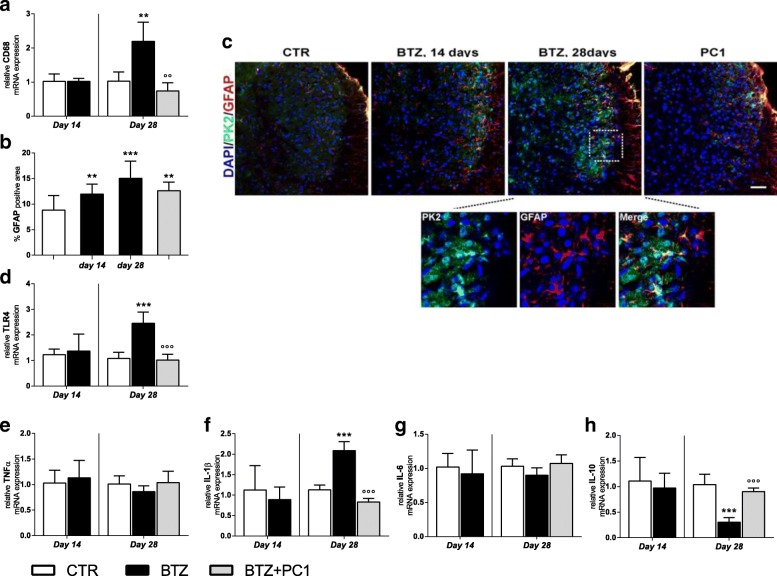
Effect of PC1 chronic treatment on neuroimmune activation in the lumbar spinal cord. **a** mRNA levels of CD68. **b** the percentage of GFAP positive area in the spinal cord sections of CTR, BTZ 14 days (day 14 corresponding to c.d. 2.4 mg/kg), BTZ 28 days (day 28, corresponding to c.d. 4.8 mg/kg), and BTZ + PC1 mice. Quantitative analysis of GFAP positive signal (**b**) was computed as integrated optical density for arbitrary areas (6 sections per animal, 5 animals). Immunofluorescence double staining (**c**) shows the colocalization (yellow) of PK2 (green) with GFAP (astrocytes, red) in the spinal cord of CTR, BTZ 14 days, BTZ 28 days, and PC1 (BTZ + PC1) mice. Cell nuclei were counterstained with DAPI (blue fluorescence), statistical analysis was performed by means of one-way ANOVA analysis of variance followed by Bonferroni’ s post-test. **p < 0.05, ***p < 0.01 vs CTR. **d**–**g** mRNA levels of TLR4 and of the pro-inflammatory cytokines TNF-α, IL-1β, and IL-6 respectively while **h** reports the levels of the anti-inflammatory cytokine IL-10. All the measurements were performed 14 days after the first BTZ administration, before starting the PC1 treatment (CTR and BTZ groups), and at the end of the BTZ/BTZ + PC1 protocol (CTR, BTZ, BTZ + PC1 groups). mRNA levels, determined by real-time qPCR, were expressed in relation to GAPDH and presented as fold increases over the levels of CTR animals (at the same time point). (**a**, **d**–**h**) Data represent mean ± SD of 6 mice/group. At day 14, statistical analysis was performed by means of *t* test while at day 28 by means of one-way ANOVA followed by Bonferroni’s post-test. ***p < 0.001 vs CTR; °°°*p* < 0.001 vs BTZ day 28

As illustrated in Fig. 7, the spinal cord was marginally affected by the lower cumulative BTZ dose: in fact, at day 14, we only observed an increase of GFAP immunofluorescence signal: panel c reports a representative image and panel b its quantification as percentage of GFAP positive area (**p < 0.01 vs CTR). No other significant change was present. Increasing the cumulative dose of BTZ, at day 28, we observed an upregulation of CD68 (panel a; **p < 0.01 vs CTR) and TLR4 mRNA (panel d; ***p < 0.001 vs CTR). These alterations were concurrent to increased levels of IL-1β (panel f; ***p < 0.001 vs CTR) and decreased levels of the anti-inflammatory cytokine IL-10 (panel h; ***p < 0.001 vs CTR). No alterations in mRNA levels of TNF-α (panel e) and IL-6 (panel g) were detected. In this tissue, as shown in panel c, immunofluorescence double-staining images illustrate that PK2 signal is associated with GFAP. PC1 was able to counteract the immune/glial activation in the lumbar spinal maintaining low levels of CD68 and TLR4 (panels a, °°*p* < 0.01 vs BTZ day 28, and d °°°*p* < 0.001 vs BTZ day 28), preserving a correct pro-/anti-inflammatory cytokine balance (panels f and h; °°°*p* < 0.001 vs BTZ day 28) and opposing to a dose-related GFAP increase (panel b and c). However, confirming the decrease of PK2 signal after PC1 treatment (Fig. 6), also PK2/GFAP colocalization disappears in PC1-treated animals (Fig. 7c).

##### Morphological studies: electron microscopy evaluations

Ultrastructural examination of DRG was performed to assess a protective role of the PK antagonist on a progressive neuronal damage due to BTZ cumulative dose.

**Fig. 8:**
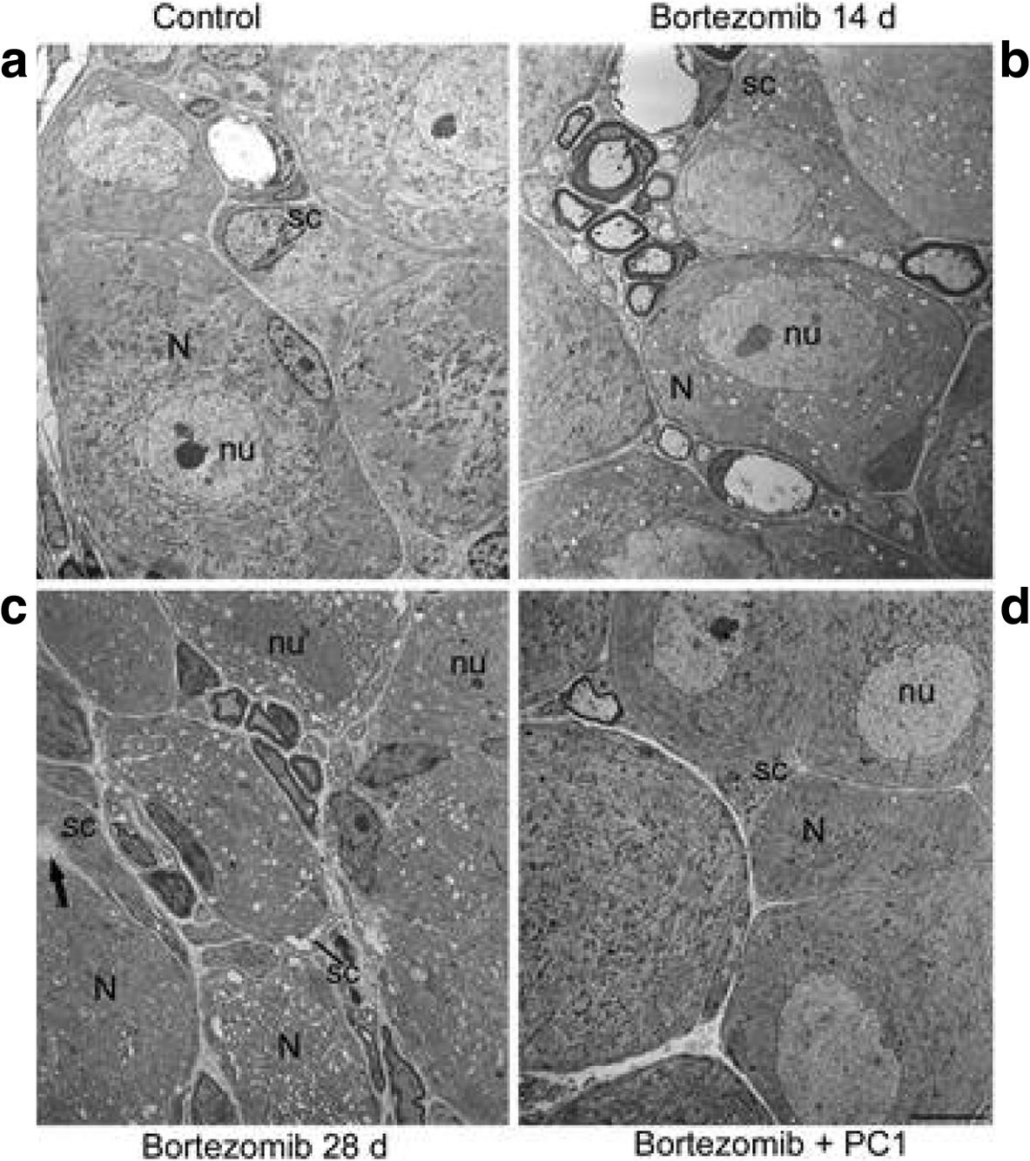
Morphological evaluation of DRG. As shown in **a** no morphological alterations are evident in both nerve cell bodies (N) and satellite glial cell (sc) sheaths of CTR mice. nu, nerve cell body nucleus. After 14 days of bortezomib, neurons (N) and satellite glial cells (sc) exhibit swollen mitochondria intermingled with some mitochondria of normal morphological features. nu, nerve cell body nucleus (**b**). At the end of BTZ treatment (day 28), severe morphological abnormalities like swollen mitochondria and enlarged endoplasmic reticulum cisternae scattered within the cytoplasm of both nerve cell bodies (N) and satellite glial cells (sc) can be seen (**c**). Some neurons show a rather dark nucleus (nu). Arrow points to a satellite glial cell (sc) sheath partially detached from the nerve cell body (N). In bortezomib + PC1 group (**d**), neurons (N) and satellite glial cells (sc) appear quite preserved, although some clear vacuoles scattered in the cytoplasm are still present. Bar = 5 μm (also applies to **a**–**c**)

Considering DRG, in physiological conditions, each nerve cell body is usually enveloped by a satellite glial cell sheaths. As illustrated in the electron microscopy images, ultrastructural examination of the control ganglia (Fig. 8a) confirmed the above described organization, and in all cells of the DRG, there was no evidence of morphological alterations. Animals subjected to BTZ administration already at day 14 showed some ultrastructural alterations; in particular, some satellite glial cell sheaths were partially detached from their own enveloped nerve cell bodies. Moreover, a greater number of neurons and satellite glial cells exhibited swollen mitochondria intermingled with some mitochondria of normal morphological feature (panel b). Increasing the cumulative dose, 28 days after the first BTZ administration, the ultrastructural examination of DRG (Fig. 8c) showed that in many cases, the satellite glial cell sheaths were detached from the nerve cell bodies. Both the two cell types showed formation of clear vacuolization scattered within the cytoplasm. Some of these vacuoles were due to swollen mitochondria, while the largest flattened and membrane-limited structures corresponded to enlarged endoplasmic reticulum cisternae. Some neurons had a rather dark nucleus instead of a normally euchromatic one. After PC1 administration, most neurons and satellite glial cells appeared well preserved, even if it is evident that some nerve cell bodies and satellite glial cells still showed some clear vacuoles scattered in the cytoplasm (Fig. 8d).

##### PK2 measurement in serum

**Fig. 9:**
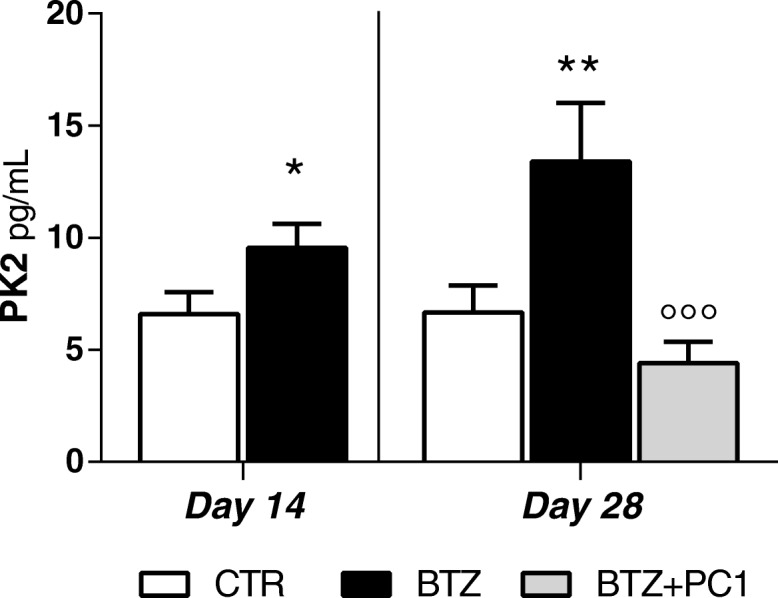
PK2 serum levels. PK2 serum levels were measured by ELISA 14 days after the first BTZ administration, before starting the PC1 treatment (CTR and BTZ groups), and at the end of the BTZ/BTZ + PC1 protocol (CTR, BTZ, BTZ + PC1 groups). Data represent mean ± SD of 6 mice/group. At day 14, statistical analysis was performed by means of *t* test while at day 28 by means of one-way ANOVA followed by Bonferroni’s post-test.*p < 0.05, **p < 0.01 vs CTR; °°°*p* < 0.001 vs BTZ day 28

Figure 9 shows PK2 protein levels measured in the serum at day 14 (before starting PC1 treatment) and at the end of the chemotherapeutic/PC1 treatment (day 28). In BTZ-treated mice, an increase of serum PK2 levels was observable already 14 days after the first BTZ injection (*p < 0.05 vs CTR). PK2 levels were still high in BTZ mice at day 28 (**p < 0.01 vs CTR). PC1 treatment was able to completely normalize altered serum PK2 levels (°°°*p* < 0.001 vs BTZ day 28).

##### Effect of PK antagonism on hypersensitivity during repeated cycles of BTZ

**Fig. 10:**
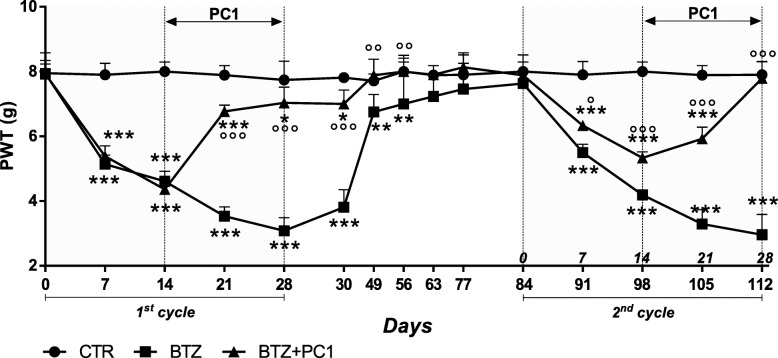
Effect of PK antagonism on mechanical allodynia during repeated cycles of BTZ. Following the interruption of a classical BTZ protocol of 28 days (BTZ 0.4 mg/kg 3 times week/4 weeks) and PC1 (s.c. 150 μg/kg twice day) chronic treatment (from BTZ day 14 to 28), mice progressively recovered from BIPN. At day 84, in the presence of basal mechanical thresholds, mice that have been previously treated with the chemotherapeutic drug (first BTZ cycle) underwent a second identical treatment with BTZ (BTZ 0.4 mg/kg, 3 times week/4 weeks). At day 98, mice previously treated with PC1 started a new chronic treatment with the antagonist. All animals were monitored until the end of the second BTZ and PC1 treatment (28 days since the beginning of the second cycle corresponding to 112 days after the first BTZ injection). Data represent mean ± SD of 6 mice/group. Statistical analysis was performed by mean of two-way ANOVA followed by Bonferroni’s post-test. *p < 0.05, **p < 0.01, ***p < 0.001 vs vehicle/CTR; °*p* < 0.05, °°*p* < 0.01, °°°*p* < 0.001 vs BTZ

As shown in Fig. 10, after the suspension of a first BTZ cycle of the duration of 28 days, mice were monitored over time until they completely recovered, returning to their basal mechanical thresholds. At day 84, animals started a new BTZ treatment with a schedule that was identical to the one used for the first BTZ cycle. As illustrated in the figure, a second BTZ cycle induces in mice an allodynic effect similar to that observed in the first BTZ cycle. However, in mice previously treated with PC1 (first cycle), the allodynic effect induced by BTZ was less intense in comparison with that observed in BTZ-only re-treated mice (°*p* < 0.05 and °°°*p* < 0.001 vs BTZ at day 7 and 14 respectively). In addition, a second PC1 chronic treatment (14 days duration) was able to completely reverse allodynia (°°°*p* < 0.001 vs BTZ day 28).

## Discussion

In this paper, we describe for the first time the role of prokineticin (PK) system in the development and progression of bortezomib (BTZ)-induced peripheral neuropathy (BIPN) that represents one of the main dose-limiting side effects in BTZ therapy, and we demonstrate a protective role of the PK antagonist PC1 on the pathology progression. Prokineticins (PKs) belong to a novel family of chemokines and are now recognized as important regulators at cross road of inflammation and pain [13, 14]. PK2 can induce a pro-inflammatory macrophage phenotype [12] and nociceptive sensitization [27] and are involved in the development of inflammatory and pathological pain [15].

In our protocol, BIPN was induced in mice by using a BTZ dosage able to induce a detectable neuropathic phenotype limiting systemic side effects [22]. To assess the role of PK system in BIPN development, we performed biochemical and electron microscopy analysis at two different time points: 14 days after the first BTZ injection, before starting PC1 treatment, and at the end of chemotherapeutic schedule (day 28). Therapeutic treatment with the PK antagonist PC1 was started after verifying in mice the presence of hypersensitivity.

Our behavioral evaluations showed that BTZ was able to induce in mice a dose-related neuropathy characterized by the presence of allodynia and hyperalgesia. Preclinical data have demonstrated that antineoplastic drugs can activate both innate and adaptive immune responses as well as peripheral and central neuronal accessory cells like satellite cells, Schwann cells, astrocytes, and microglia [4, 28]. In particular, chemotherapeutics can cross the blood-nerve barrier, accumulating in dorsal root ganglia and in the peripheral nerves and exerting a toxic action with consequent immune cell infiltration and activation [8]. Consistently, our biochemical and immunofluorescence results indicate that after 14 days of BTZ, in the presence of an hyperalgesic and allodynic state, PNS stations are characterized by increased levels of macrophage activation markers, i.e., CD68 and TLR4, and by the presence of a pro-inflammatory cytokine profile due to high levels of the investigated pro-inflammatory cytokines (TNF-α, IL-1β, and IL-6) and low levels of the anti-inflammatory cytokine IL-10 which is particularly evident in DRG. Moreover, our electron microscopy evaluations demonstrated that DRG of BTZ mice are characterized by ultrastructural abnormalities like the presence of some partially detached satellite cell sheaths and some swollen mitochondria in neurons and satellite cells. At this time point, we only observed an increase of GFAP in the spinal cord without measuring any other biochemical alteration. Our results showing an increase of CD68 marker and of TLR4 expression in the peripheral nervous tissues are consistent with the recent literature that suggests the importance of macrophage infiltration and activation in PNS in CIPN development [8]. For example, it has been recently demonstrated that the intravenous immunoglobulin administration was able to reduce or prevent BTZ-induced heat and mechanical allodynia in rat by decreasing or preventing M1 macrophage infiltration [10] in the peripheral nerves. In our study, by increasing the BTZ cumulative dose (day 28), we observed a further lowering of the response thresholds to mechanical and thermal stimuli in BTZ-treated animals. The increased hypersensitivity correlates with a more severe structural damage in DRG and with the appearance of a more pronounced neuroinflammatory condition also evident at the spinal cord level. Interestingly, after 28 days of BTZ treatment, we also observe an overexpression of PK system in all the tissues involved in pain transmission (sciatic nerve, DRG, spinal cord). Immunofluorescence data suggest that in PNS at the higher BTZ dose, CD68 + cells co-express PK2. Therefore, we can suppose that infiltrating activated macrophages may represent an important source of PK2 in DRG and sciatic nerve even if it appears evident that other cell types like satellite cells and neurons may contribute to PK2 increase. In our paradigm, the activation of the PK system in BIPN is delayed in comparison with painful symptoms and the precocious neuroinflammation. This later PK activation was somehow surprising, since in previous work from ours [13, 14] and other groups, PK system [20] activation well correlated with the development of hypersensitivity. Here, we demonstrated that in BIPN, this chemokine family has however an important role in sustaining, maintaining, and worsening hypersensitivity, neuroinflammation, and structural damage of DRG. In fact, chronic treatment with PC1, even if it was started in the presence of an established hypersensitivity, was able to counteract the further decrease of mechanical and thermal thresholds, to preserve against the neurotoxic damage to DRG and to reverse the established neuroinflammation, rebalancing pro- and anti-inflammatory cytokines in sciatic nerve and DRG. We can assume that during BTZ treatment, infiltrated and resident-activated immune cells, in association with satellite cells and Schwann cells, produce pro-inflammatory cytokines leading to a further recruitment of immune cells into damaged nervous tissues. These infiltrating macrophages not only produce PK2 but also express PK-Rs receptors [26]; hence, PK2 can act in autocrine or paracrine way sustaining a neuroinflammatory loop that exacerbate the neuronal damage and sustain a progressive glial activation at the spinal cord level [20]. A possible signaling pathway could be the one suggested by the group of Qu et al. [28]. Authors demonstrated that STAT3 signaling plays a crucial role in PK2 regulation and that phosphorylated STAT3 can directly bind to the Pk2 promoter. Furthermore, a recent study [29] demonstrated that phosphorylated STAT3 levels were significantly increased after BTZ administration and that STAT3 activation in DRG contributes to BIPN. On the basis of these data, we can speculate that the activation of STAT3, consequent to pro-inflammatory cytokine increase in the peripheral nervous stations [30], could be one of the mechanisms involved in the PK2 upregulation. The effect of PC1 may be in part related to its ability to reduce macrophage activation and infiltration in PNS and to prevent PK system upregulation that plays a crucial role in prompting spinal cord neuroinflammation. In addition, as also supported by the acute antiallodinic effect of PC1, PKs can also act on PK-Rs expressed by neurons and glial cells enhancing pain pathway transmission [17] which also occurs through TRPV1 sensitization [31, 32]. Our results also confirm the importance of astrocytes in CIPN [33]. In fact, in our experiments, GFAP is the only marker that we find precociously activated in the spinal cord. It was recently suggested that the presence of mechanical hypersensitivity due to BTZ treatment correlated to an upregulation of GFAP [34, 35], and more recently, Salvemini’s group described that the development of BIPN is lost when S1PR1 (sphingosine-1-phosphate receptor 1) is deleted in astrocytes, suggesting a central role of astrocytes in sustaining CNS sensitization [33]. Interestingly, by blocking the activation of the PK system with PC1, we prevent a further neuroinflammatory condition in the spinal cord. In BTZ + PC1-treated animals, in fact, we did not detect any increase of glial activation markers CD68 and TLR4 that are indeed significantly enhanced in BIPN animals after 28 days of BTZ treatment. As already observed in other experimental models, IL-1 and IL-10 appear to be the main cytokines modulated in the spinal cord in the presence of a neuropathic state and the treatment with PC1 is able to prevent the IL-1/IL-10 unbalance. We can therefore hypothesize that in the spinal cord, there is an early activation of astrocytes that is independent from the PK system. Astrocytes start to produce PK2, as demonstrated by the colocalization of PK2 and GFAP signals in the immunofluorescence experiments and confirming what we already observed in the CCI model [14]. PK2 promotes microglia activation and cytokine alterations that may participate in central sensitization; antagonizing the PK system prevents this later activation. The precocious astrocyte activation is not completely reverted by the PK antagonism and may be responsible for the only partial anti- hyperalgesic effect observed in PC1 mice. We must however underline that in PC1-treated mice, we did not observe a BTZ dose-related increase of GFAP signal, suggesting that blocking PK2 may be useful to control astrogliosis. Moreover, our data support the well-known flow of neuroimmune activation from the periphery to the central nervous system [36–38] at the basis of the development of pathological pain and underline the role of the prokineticin system in this sensitization process.

In this study, we also measured circulating levels of PK2. PK2 is a secreted protein, and elevated levels of the chemokine have been reported in serum from mice with experimental EAE autoimmune disease [39] as well as in multiple sclerosis patients. In BTZ-treated mice, we also found significant elevated levels of PK2 already after 14 days of BTZ treatment, therefore preceding the PK2 activation in nervous tissues. We can speculate that peripheral leukocytes may be the main source of the chemokine, since monocytes, granulocytes, and lymphocytes produce and release PK2 when activated [11, 16, 40]. The cytotoxic action exerted by BTZ and the presence of a neuroinflammatory condition in PNS stations could represent an activation signal for peripheral immune cells. However, further experiments are needed in order to understand the source of PK2 in blood from BIPN animals.

Finally, the data reported in this study could have translational implications. First of all, considering that BIPN develops in about 1/3 of BTZ-treated patients, PC1 may be administered only when the symptoms have appeared allowing patients continue the chemotherapeutic treatment. In addition, we show that PC1 has a protective role in a two-cycle BTZ schedule: in fact, in the second cycle, the allodynic effect promoted by BTZ is less evident in mice previously treated with PC1 if compared to that observed in BTZ-only re-treated mice. Furthermore, the second PC1 treatment completely normalizes the mechanical thresholds. Considering that patients often undergo multiple chemotherapeutic cycles, the protective role exerted by PC1 on a second chemotherapeutic cycle could be clinically relevant in order to slow down the re-appearance of the side effects. We plan to deeply investigate the reason behind this protective role of PC1 in future studies. At the moment, we can only speculate that the protective effect exerted by PC1 could be due to its ability to counteract neuroinflammation and more likely to its protective role on DRG ultrastructure. It can be hypothesized that chronic PC1 treatment may induce long-lasting modification in the PK system or enhance protective mechanisms that may be important in a second BTZ cycle, but further experiments are needed to sustain this possibility.

## Conclusions

In conclusion, this work indicates the PK system as a strategical pharmacological target to counteract the progression of BTZ-induced peripheral neuropathy. Blocking PK2 activity reduces progressive BTZ toxicity in the DRG, reducing neuroinflammation and structural damage, and may prevent spinal cord sensitization. Considering that the development of CIPN seems to be independent from the primary mechanism of action of the antitumoral drug [32], it could be important to verify if the PK antagonism could be efficacious also to contrast peripheral neuropathy which follows the treatment with other chemotherapeutics.

## Abbreviations

BIPN: Bortezomib-induced neuropathy
BTZ: Bortezomib
CIPN: Chemotherapy-induced peripheral neuropathy
DRG: Dorsal root ganglia
PK: Prokineticin
PK-R: Prokineticin receptor
TRPA: Transient receptor potential ankyrin
TRPV: Transient receptor potential vanilloid
